# Supplementary material for: ProteinShader: illustrative rendering of macromolecules
Source: BMC Struct Biol. 2009 Mar 30;9:19. doi: 10.1186/1472-6807-9-19 (PMC2672931; doi:10.1186/1472-6807-9-19)
Supplement: Additional file 1 — ProteinShader program without source code. This compressed file contains the complete ProteinShader program including associated libraries, but no source code. A README.txt file gives an overview of the ProteinShader distribution, and the index.html file in the help subdirectory has directions on getting started with the program as well as a set of tutorials. [file 1472-6807-9-19-S1.zip › ProteinShader-beta-0_9_4-binary/help/api/org/proteinshader/gui/viewing/Lighting.html]

Lighting (ProteinShader API)


|  |  |  |
| --- | --- | --- |
| **Overview** \| **Package** \| **Class** \| **Use** \| **Tree** \| **Deprecated** \| **Index** \| **Help** | |  |
| **PREV CLASS**   **NEXT CLASS** | **FRAMES**    **NO FRAMES**     **All Classes** |  |
| SUMMARY: NESTED \| FIELD \| CONSTR \| METHOD | DETAIL: FIELD \| CONSTR \| METHOD |  |


---


## org.proteinshader.gui.viewing Class Lighting

```
java.lang.Object
  org.proteinshader.gui.viewing.Lighting
```

---

``` public class Lighting extends Object ```

Sets up OpenGL lighting for the Renderer.

---

| **Field Summary** | |
| --- | --- |
| `static float[]` | `AMBIENT_LIGHT`             The ambient light is { 0.2f, 0.2f, 0.2f, 0.0f }. |
| `static float[]` | `DIFFUSE_LIGHT`             The diffuse light is { 0.95f, 0.95f, 0.95f, 0.0f }. |
| `static float[]` | `LIGHT0_POSITION`             The position of light zero is {200.0f, 200.0f, 200.0f, 1.0f}. |
| `static float[]` | `SPECULAR_LIGHT`             The specular light is { 0.95f, 0.95f, 0.95f, 0.0f }. |


**Constructor Summary**

`Lighting()`
  
          Constructs a Lighting object.


| **Method Summary** | |
| --- | --- |
| `void` | `disableLighting(GL gl)`             Disables the lighting. |
| `void` | `enableLightOne(GL gl, float x, float y, float z)`             Light one is set to the xyz-coordinates given as arguments |
| `void` | `enableLightZero(GL gl)`             Light zero is set to the position given in LIGHT0\_POSITIONS. |

**Methods inherited from class java.lang.Object**

`clone, equals, finalize, getClass, hashCode, notify, notifyAll, toString, wait, wait, wait`

**Field Detail**

### LIGHT0\_POSITION

```
public static final float[] LIGHT0_POSITION
```

:   The position of light zero is {200.0f, 200.0f, 200.0f, 1.0f}.
    If the last element in the array is zero, then the light is
    directional. If the last element is non-zero, the light is
    positional.

---


### AMBIENT\_LIGHT

```
public static final float[] AMBIENT_LIGHT
```

:   The ambient light is { 0.2f, 0.2f, 0.2f, 0.0f }.

---


### DIFFUSE\_LIGHT

```
public static final float[] DIFFUSE_LIGHT
```

:   The diffuse light is { 0.95f, 0.95f, 0.95f, 0.0f }.

---


### SPECULAR\_LIGHT

```
public static final float[] SPECULAR_LIGHT
```

:   The specular light is { 0.95f, 0.95f, 0.95f, 0.0f }.


**Constructor Detail**

### Lighting

```
public Lighting()
```

:   Constructs a Lighting object.


**Method Detail**

### enableLightZero

```
public void enableLightZero(GL gl)
```

:   Light zero is set to the position given in LIGHT0\_POSITIONS.

---


### enableLightOne

```
public void enableLightOne(GL gl,
                           float x,
                           float y,
                           float z)
```

:   Light one is set to the xyz-coordinates given as arguments

---


### disableLighting

```
public void disableLighting(GL gl)
```

:   Disables the lighting.

    :   **Parameters:**: `gl` - the current GL object.


---


|  |  |  |
| --- | --- | --- |
| **Overview** \| **Package** \| **Class** \| **Use** \| **Tree** \| **Deprecated** \| **Index** \| **Help** | |  |
| **PREV CLASS**   **NEXT CLASS** | **FRAMES**    **NO FRAMES**     **All Classes** |  |
| SUMMARY: NESTED \| FIELD \| CONSTR \| METHOD | DETAIL: FIELD \| CONSTR \| METHOD |  |


---

# *Copyright © 2007-2008*
